# Supplementary material for: Experience and coping strategies of bowel dysfunction in postoperative patients with rectal cancer: a systematic review of qualitative evidence
Source: PeerJ. 2023 Mar 24;11:e15037. doi: 10.7717/peerj.15037 (PMC10042155; doi:10.7717/peerj.15037)
Supplement: Supplemental Information 2 [file peerj-11-15037-s002.docx]

1. The rationale for conducting the systematic review / meta-analysis;

Due to the changes of bowel physiological structure and functional disorders after rectal cancer surgery, patients will face many bowel dysfunction for a long time, which will greatly affect their quality of life. The purpose of this review is to integrate the qualitative research on the experience of bowel dysfunction and coping strategies in postoperative patients with rectal cancer.

Although the postoperative bowel symptoms of rectal cancer can be evaluated by objective indicators, individual differences may affect the understanding of intestinal symptoms. Qualitative research is based on in-depth mining of small sample groups to obtain information, the validity and extensibility of single research results are still limited. All aspects of postoperative bowel symptom experience need to be integrated based on multiple qualitative studies to form stronger evidence. Our purpose is to describe the perioperative experience and needs of patients with rectal cancer experiencing bowel dysfunction, and summarize their feelings and responses by integrating relevant qualitative studies. The results of this study can provide a reference for nurses to formulate practical measures to implement bowel function management.

1. The contribution that it makes to knowledge in light of previously published related reports, including other meta-analyses and systematic reviews; e.g. _________

To describe the perioperative experience and needs of patients with rectal cancer experiencing bowel dysfunction, and summarize their feelings and responses by integrating relevant qualitative studies. The results of this study can provide a reference for nurses to formulate practical measures to implement bowel function management.
